# The high osmotic response and cell wall integrity pathways cooperate to regulate morphology, microsclerotia development, and virulence in *Metarhizium rileyi*

**DOI:** 10.1038/srep38765

**Published:** 2016-12-12

**Authors:** Zhangyong Song, Qiang Zhong, Youping Yin, Ling Shen, Yan Li, Zhongkang Wang

**Affiliations:** 1 Chongqing Engineering Research Center for Fungal Insecticide, School of Life Science, Chongqing University, Chongqing 400030, People’s Republic of China

## Abstract

Microsclerotia (MS) formation was successfully induced in *Metarhizium rileyi* under changing liquid culture conditions. Mitogen-activated protein kinases (MAPKs) play important roles in fungal development and in coordinating many stress responses. To investigate how *M. rileyi* transduces growth stress and regulates MS differentiation, we characterized the roles of two MAPKs, Hog1- and Slt2-type orthologues, in *M. rileyi*. Compared with the wild-type strain, the deletion mutants of *Mrhog1* (*ΔMrhog1*) and *Mrslt2* (*ΔMrslt2*) delayed germination and vegetative growth, displayed sensitivities to various stress, and produced morphologically abnormal clones. The *ΔMrhog1* and *ΔMrslt2* mutants significantly reduced conidial (42–99%) and MS (96–99%) yields. A transcriptional analysis showed that the two MAPKs regulate MS development in a cooperative manner. Insect bioassays revealed that *ΔMrhog1* and *ΔMrslt2* had decreased virulence levels in topical (36–56%) and injection (78–93%) bioassays. Our results confirmed the roles of *MrHog1* and *MrSlt2* in sensing growth-related stress and in regulating MS differentiation.

Microsclerotia (MS) are pseudoparenchymatal aggregations of hyphae that become melanised as they develop. They have diameters of 50–600 μm and are comprised of only a few cells. They are produced by many phytopathogenic fungi because of their survival in soil and decaying plant materials. For biocontrol applications, MS have been induced in high concentrations in liquid culture. They can be used as mycoinsecticides to manage insect pests^1^,^2^, as antagonists of plant pathogenic fungi^3^, as bioherbicides for the management macrophytes^4^, or bionematicides against root knot nematodes^5^. MS are desiccation tolerant, with excellent storage stability, and have the potential to produce many infective conidia^2^,^4^. These capabilities make MS a promising fungal propagule. The realization of alternative propagules could alleviate present limitations in the production and commercialization of biocontrol fungi, such as the *Metarhizium rileyi,* which is environmentally-friendly, dimorphic entomopathogen fungus^6^,^7^,^8^.

Previously, our laboratory had realized *M. rileyi* MS formation and fermentation in a liquid amended medium (AM)^2^. To enhance the fermentation efficiency, we investigated the molecular mechanism of MS formation and found that internal oxidative stress could trigger MS differentiation^9^,^10^. We additionally investigated the role of regulatory components in H_2_O_2_ production and hypal polarized growth^11^,^12^. Then we investigated the changing culture conditions during MS development and found the important roles of Sho1p and Sln1p in *M. rileyi* MS differentiation^13^. Furthermore, we discovered that all of the genes involved in cell wall integrity (CWI) and the high osmolarity glycerol (HOG) signalling pathway were up-regulated when MS initiation in our previous comparative transcriptome analysis (data not shown)^10^. However, little is known about how *M. rileyi* transduces signals associated with changing culture conditions through the CWI and HOG signalling pathways during MS development or how the responses affect MS differentiation.

Adaptation to changing environments is crucial for fungi viability^14^. Fungi contain complex signalling pathway networks to handle with these stresses^15^. Mitogen-activated protein kinase (MAPK) signalling pathways are conserved and play important roles in sensing environmental stimuli, in transmitting extracellular signals to the nucleus to modulate gene expression, in regulating morphology, in responding to abiotic and biotic stresses, and in virulence/pathogenicity^16^,^17^,^18^. Five MAPK signal pathways in fungi are characterized and known to regulate different processes, such as mating, filamentous growth, high osmolarity responses, maintenance of cellular integrity, and ascospore formation. The HOG pathway is used to stimulate transcriptional responses to the osmolarity^14^, and CWI controls the maintenance of cell shape and integrity^17^. Although fungi have mechanisms that guarantee the specificity of MAPK signalling pathways and that insulate other pathways, the pathways respond to many stress in a coordinated manner^17^,^19^. In some fungi, the HOG and CWI pathways interact in responses the cell wall^20^,^21^,^22^ and oxidative stresses^23^,^24^.

MAPK pathways are comprised of three protein kinases: MAP kinase kinase kinase (MAPKKK), MAP kinase kinase (MAPKK), and MAP kinase (MAPK), which are conserved among the MAPK pathways. Traditionally, Hog1p, the core MAPK of the HOG pathway, is activated in sense and responds to fluctuations in environmental osmotic pressure^25^,^26^. Slt2p, the central MAPK of the CWI pathway, is activated in response to cell wall stress to maintain cell wall integrity^27^,^28^,^29^. However, the two MAPKs are not restricted to individual stimuli, but their responses can be elicited by various environmental stresses^30^,^31^,^32^. Furthermore, there is some evidence of collaboration between the two MAPKs to counteract various antifungal drugs and environmental stresses^20^,^21^,^22^,^23^,^24^,^33^. However, there is no evidence of such a role in regulating MS differentiation.

To investigated the effects of the *Mrhog1* and *Mrslt2* genes on MS formation and verify whether the two MAPKs pathway regulate MS development in a cooperative way, the *Mrhog1* and *Mrslt2* genes were cloned, based on a comparative transcriptome library^10^, in the present study. We used a homologous recombination strategy to construct disruption mutants and investigated their involvement in hyphal polarized growth, conidiation, virulence, and MS formation. These mutants appeared to have defective conidial and MS cell differentiation. Insect bioassays revealed a significant decrease in the virulence of the mutants. Thus, *Mrhog1* and *Mrslt2* play important roles in fungal development and virulence. Furthermore, the expression patterns of the genes involved in CWI and HOG signalling pathways were analysed during MS development. These results provided new information about the cooperation of *Mrhog1* and *Mrslt2* in the regulation of *M. rileyi* MS differentiation.

## Results

### Bioinformatics analysis of *Mrhog1* and *Mrslt2*

Using a transcriptome library^10^, we cloned *Mrhog1* and *Mrslt2* (GenBank Accession No.: KU950829 and KU950830, respectively). The cDNA of *Mrhog1* contained 1,077 bp, encoding 358 amino acid residues, and that of *Mrslt2* contained 1,257 bp, encoding 418 amino acids (http://expasy.org/tools/protparam.html). The *Mrhog1* and *Mrslt2* genomic DNA had eight and four introns, respectively and both contained Serine/Threonine kinase domains. The deduced amino acid sequence of Mrhog1p showed similarities with those of Hog1p (99.2% identity) in *Metarhizium album*^34^, Bbhog1p (97.5% identity) in *Beauveria bassiana*^35^, and Mhog1p (98.0% identity) in *Magnaporthe oryzae*^36^. Furthermore, the deduced amino acid sequence of Mrslt2p presented similarities with those of Slt2p (98.6% identity) in *M. robertsii*^34^ and Slt2p (94.5% identity) in *B. bassiana*^35^. A phylogenetic tree analysis demonstrated that the two genes are conserved and have the closest genetic relationships with those of *Metarhizium* spp. (Fig. S1).

### Construction of *Mrhog1* and *Mrslt2* knockout mutants

As there is no public *M. rileyi* genome, fusion primer and nested integrated PCR^37^ was used to obtain the up- and down-stream genomic DNA flanking sequences (data not shown). Using the genomic DNA of the wild-type (WT) strain CQNr01 as the template, the up- (~1.1 Kb and ~1.2 Kb) and down-stream (~1.5 Kb and ~1.3 Kb) flanking sequences of *Mrhog1* and *Mrslt2*, respectively, were obtained by PCR using primers HLF1/HLR1, HRF1/HRR1, SLF1/SLR1, and SRF1/SRR1 (Table S1), respectively. The PCR fragments were digested by restriction endonucleases and ligated into the *EcoR*I/*Xho*I and *Xba*I/*Hing*III sites of pPZP-Hph-Knock^37^. The resultant plasmids were designated as pPZP-Hph-Mrhog1 and pPZP-Hph-Mrslt2.

The *Mrhog1* and *Mrslt2* genes were disrupted using the hygromycin phosphotransferase gene (*hph*) and *Agrobacterium*-mediated transformation (Fig. S2A). There were approximately 120 and 30 hygromycin B (Hyg B)-resistant colonies harbouring the Hog1- and Slt2-replacement constructs, respectively. PCR screening confirmed that the replacement constructs were integrated by homologous recombination at the targeted loci, and three *Mrhog1* disrupted mutant strains (*ΔH13*, *ΔH74*, and *ΔH96*) and two *Mrslt2* disrupted mutant strains (*ΔS6* and *ΔS11*) were obtained. Genomic Southern hybridization was performed to further confirm four of the mutants (*ΔH13*, *ΔH74*, *ΔS6*, and *ΔS11*). As shown in Fig. S2B, all of the putative homologous knockout mutants had lost the *Mrhog1* or *Mrslt2* coding sequences. The four disrupted mutants (*ΔH13*, *ΔH74*, *ΔS6,* and *ΔS11*) were used in following experiments.

### Conidial yield and vegetative growth of *ΔMrhog1* and *ΔMrslt2*

The ability of conidia to germinate was tested on Sabouraud maltose agar fortified with 1% (w/v) yeast extract (SMAY) and is shown in Fig. S3. The germination of mutants was somewhat retarded compared with WT. After 14 h, their germination rates (41.3–42.2% for *ΔH13* and *ΔH74*; 60.2–61.3% for *ΔS6* and *ΔS11*) were significantly lower than that of WT (83.3 ± 2.5%). At 24 h, the germination rates were also different between the mutants and WT. However, at 36 h, the differences in the germination rates were no longer observed (data not shown).

**Figure 1:**
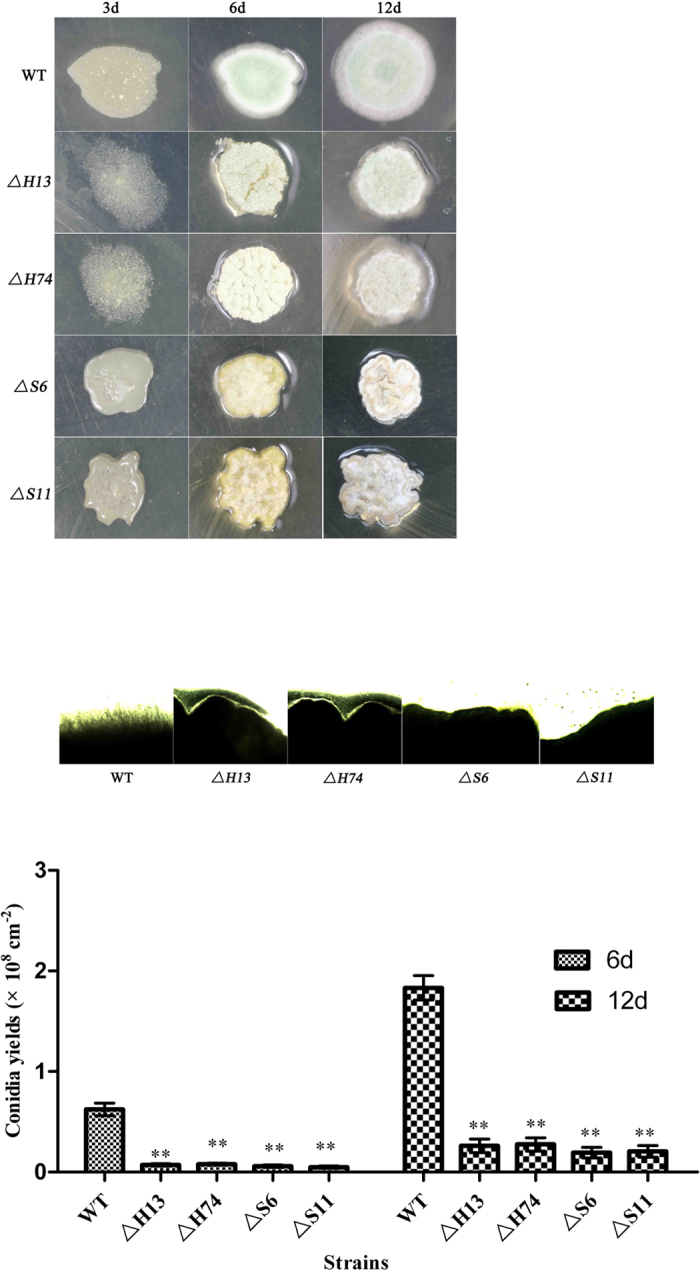
Effects of *ΔMrhog1*, *ΔMrslt2* and WT strains on growth and colony morphology. Strains were grown on SMAY medium for 3, 6, and 12 days. (**A**) Colonies on SMAY plates. In total, 3 μl of conidial suspensions (1 × 10^7^ conidia/ml) were spotted on SMAY plates and cultured under continuous light at 25 °C for 12 days. The colony morphologies were photographed without magnification. Scale bar: 0.5 cm. (**B**) Cross sections of the growth. The growing cultures were sampled at 6 days and examined under light microscopy. (**C**) Conidial yield analysis of different strains. Standard error bars indicate variation in measurements. *P < 0.05 and **P < 0.01, when compared with the WT results.

After incubation on SMAY plates, the colony morphologies were observed. There were morphological abnormalities in the mutants (Fig. 1A). At 3 days, the dimorphic switch was delayed in the mutants. After 6 days, the colony surfaces of the mutants were considerably more convoluted compared with the normal smooth colony surfaces of the WT. Furthermore, the mycelial growth of the mutants was restricted (Fig. 1B). After 12 days, the induced convolution continues, especially in *ΔMrslt2* strains. Additionally, the mutants had significantly reduced conidial yields compared with that of WT (Fig. 1C).

**Figure 2:**
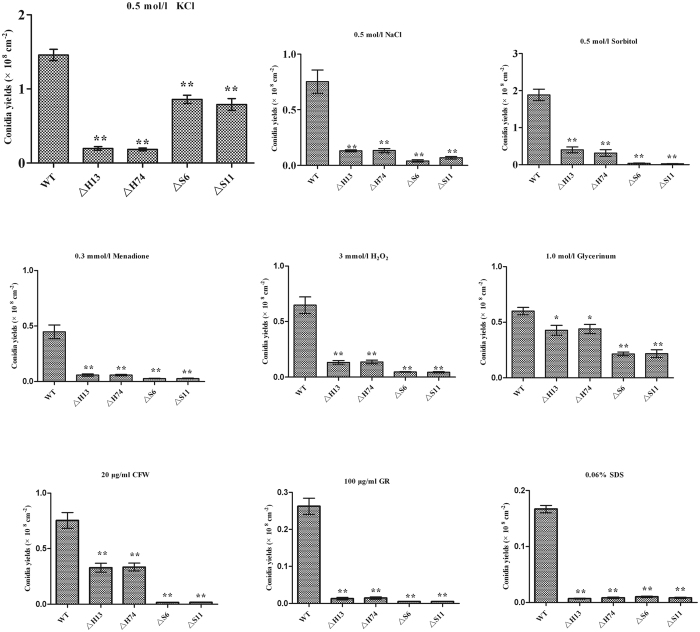
Conidial yield analysis of different strains under various non-biotic stresses. Standard error bars indicate variation in measurements. *P < 0.05 and **P < 0.01, when compared with WT results.

To examine whether the mutant strains were defective in response to abiotic stress, the tested strains were analysed on SMAY plates containing salt, osmotic, cell, and oxidative stress agents. Under the various stress conditions, the conidial yields of the mutant strains were severely affected, exhibiting 41.9–99.1% decreases, which indicated the high sensitivity of the mutant (Fig. 2). Furthermore, the restricted mycelial growth and convoluted colony surfaces were present on these stress-inducing media (data not shown).

### Fungal growth of *ΔMrhog1* and *ΔMrslt2* in liquid culture

**Figure 3:**
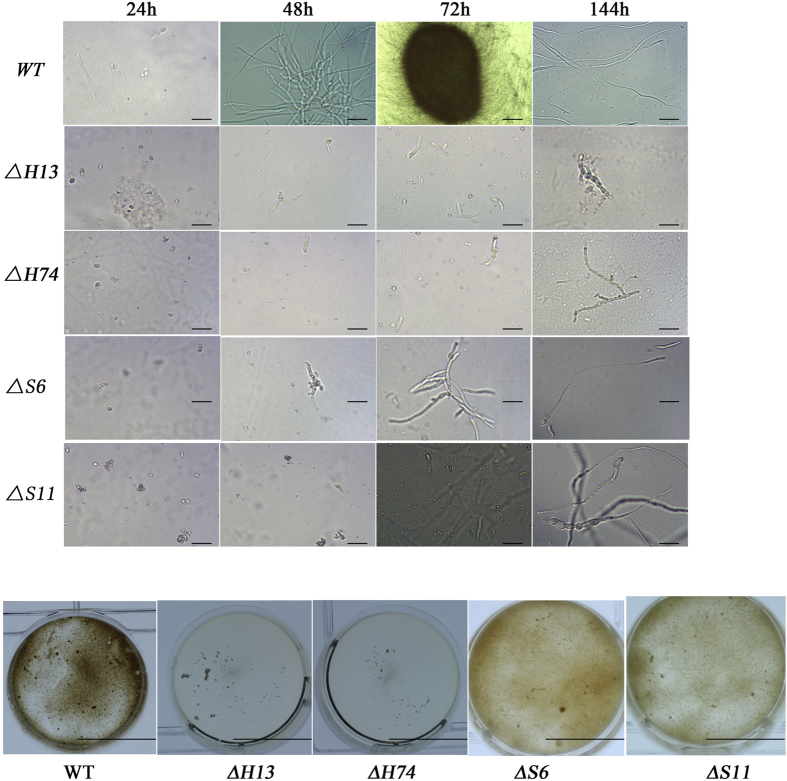
MS development in the *ΔMrhog1*, *ΔMrslt2* and WT strains. (**A**) Development of *in vitro* MS. The AM culture was inoculated with a conidial suspension from an indicated strain and incubated for 6 days at 25 °C. The growing cultures were sampled and examined under light microscopy at 24 h, 48 h, 72 h, and 144 h, respectively. Scale bar: 50 μm. (**B**) Morphological phenotypes of MS mutants in AM. Scale bar: 1 cm.

The tested strains were cultured in liquid AM according to previous methods^13^. The microscopic observations of conidial germination, hyphal growth, and MS formation are shown in Fig. 3. In 24 h of cultivation, the mutants had slower germination rates (Fig. 3A). After 48 h of vegetative growth, the WT strain started to form MS, however, germinated conidia were observed in the mutants. After 144 h, the MS produced from the WT strain, had matured and was accompanied by secondary mycelial growth, whereas there were few radial growths in the mutant cultures and abnormal structures were observed. The hyphae of *ΔMrslt2* mutants were inflated, particularly at the hyphal tips (Fig. S4). Additionally, compared with the WT, the fermentation broth of *ΔMrhog1* exhibited a lower viscosity and a higher transparency, while the *ΔMrslt2* mutants exhibited lower degree of pigmentation (Fig. 3B). The biomass of *ΔMrhog1* was reduced approximately 70% in the AM culture and 42% in the minimal medium (MM) culture (AM without basal salt sources), whereas the *ΔMrslt2* was reduced 43% in the AM culture and 22% in the MM culture (Table 1). Furthermore, the MS yields of *ΔMrhog1* and *ΔMrslt2* were reduced by approximately 99% and 96%, respectively.

### Transcriptional level analysis

**Figure 4:**
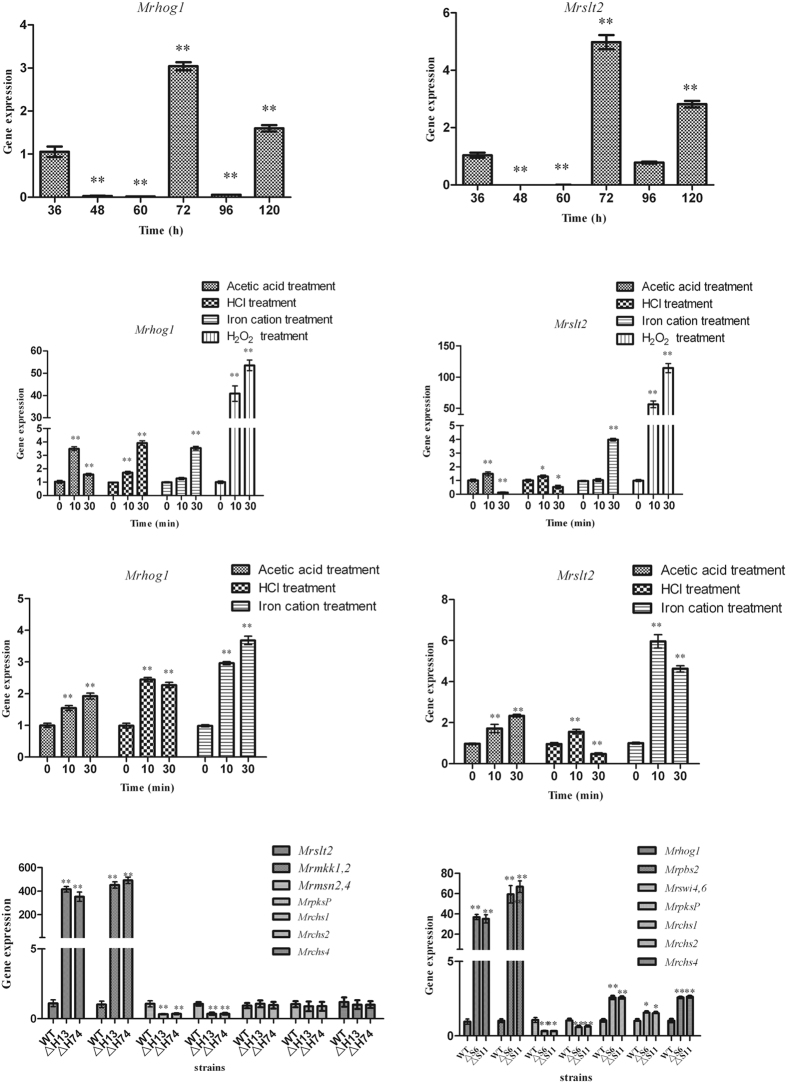
RT-qPCR analysis of gene expression levels. Relative expression levels of *Mrhog1* (**A**) and *Mrslt2* (**B**) during MS development. Relative expression levels of *Mrhog1* (**C**) and *Mrslt2* (**D**) following independent treatments with acetic acid, HCl, H_2_O_2_, and iron cation in AM. Relative expression levels of *Mrhog1* (**E**) and *Mrslt2* (**F**) following independent treatments with acetic acid, HCl, and iron cation in MM. Relative expression levels of genes in *ΔMrhog1* (**G**) and *ΔMrslt2* (**H**). Standard error bars indicate variations in measurements. *P < 0.05 and **P < 0.01, when compared with the results observed at 36 h and 0 min, respectively.

The expression levels of *Mrhog1* and *Mrslt2* during MS development were analysed. When compared with the transcriptional levels at the germinating spore stage, the transcriptional levels of *Mrhog1* and *Mrslt2* peaked when MS initiation occurred (72 h) (Fig. 4A and B), indicating that the expression of *Mrhog1* and *Mrslt2* may be involved in regulating MS formation.

*Mrhog1* and *Mrslt2* were up-regulated when the AM culture was amended with acetic acid, HCl, H_2_O_2_, and iron cations. In particular, the expression levels of the two genes were sharply up-regulated, 10 min after the addition of H_2_O_2_ (Fig. 4C and D). To further analyse the expression levels of genes under changing culture conditions, we used in the MM culture. The results were the same as in the AM investigation (Fig. 4E and F). This indicated that *Mrhog1* and *Mrslt2* were up-regulated under changing culture conditions.

To investigate whether the cooperation of *Mrhog1* and *Mrslt2* was required to regulate MS differentiation, the genes of up- and down-stream in the two signalling pathway were analysed. The genes of the CWI signalling pathway were significantly up-regulated, whereas the expression of *Mrmsn*_*2,4*_, downstream of *Mrhog1*, was significantly reduced in the *ΔMrhog1* mutants (Fig. 4G). In the *ΔMrslt2* mutants, the genes of the HOG signalling pathway were significantly up-regulated, whereas the expression of gene *Mrswi*_*4,6*_, downstream of *Mrslt2*, was significantly reduced (Fig. 4H).

The reduced pigmentation in *ΔMrhog1* and *ΔMrslt2* mutants suggested that melanin biosynthesis might be compromised. We thus analysed the transcript levels of the *MrpksP* gene, which is involved in melanin biosynthesis. Consistent with reduced pigmentation, the expression of *MrpksP* was significantly reduced in the *ΔMrhog1* and *ΔMrslt2* mutants (Fig. 4G and H). To evaluate whether the abnormal hyphal phenotypes of the mutants were due to changes in cell wall structure, we examined the transcription levels of three chitin synthase genes (class I, II, and IV chitin synthases, *Mrchs1*, *Mrchs2*, and *Mrchs4*, respectively). The results suggested that the expression levels of these genes were significantly increased in the *ΔMrslt2* mutants, however, no expression changes were observed in *ΔMrhog1* mutants (Fig. 4G and H).

### *Mrhog1* and *Mrslt2* influenced the virulence of *M. rileyi*

**Figure 5:**
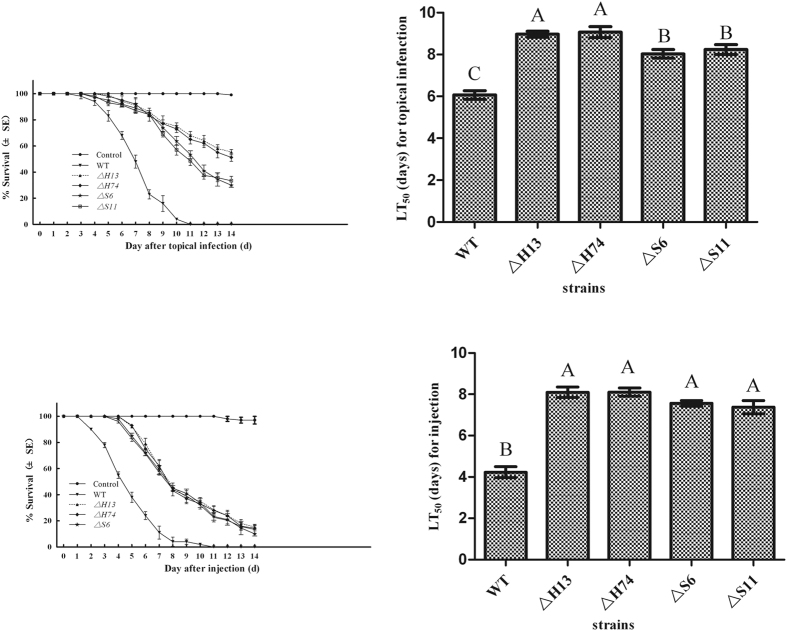
Virulence bioassays. Survival trends of insect after topical application (A) and directly injection (C) of tested strains. The median lethal time (LT_50_) values for topical application (B) and directly injection (D) assays. For the blank control, 5 μl of pure cotton seed oil alone or sterile water with 0.01% Tween 80 was applied to or injected into the larvae, respectively. Error bars are standard error of three trials. Mean values followed by different letters are significantly different (Duncan’s multiple range tests).

Mortality was measured over a 14-day period, and the results showed that the mortality rate increased with the post-inoculation time (Fig. 5). The mutants were significantly less virulent than the WT. The mean lethal time 50 (LT_50_) values were 6.1 ± 0.3 days in the topical bioassay and 4.2 ± 0.4 days in the injection bioassays for WT, whereas the LT_50_ values of *ΔMrhog1* and *ΔMrslt2* mutants increased to 9.5 ± 0.2 and 8.3 ± 0.3 days, respectively, in the topical bioassay, 8.1 ± 0.3 and 7.5 ± 0.2 days, respectively, in the injection bioassays.

## Discussion

In this study, using the transformation protocol recently developed in our laboratory^38^, we examined the functions of the Hog1- and Slt2-type MAPKs in *M. rileyi* in response to stress, and their roles in morphology and pathogenicity. Consistent with findings in other fungi, two MAPKs are important in developmental processes and in pathogenesis^26^,^28^,^39^,^40^. Moreover, our current work indicated that the two MAPKs regulate MS development in a cooperative manner.

The *M. rileyi* exhibits a dimorphic life style and the yeast-to-hyphae transition is a multi-step process^41^,^42^. To sense the signal, fungi may use several signalling pathways to coordinate the appropriate cellular actions^14^. The Hog1 and Slt2 kinases are various signal-activated proteins. Intriguingly, the inactivation of *Mrhog1* or *Mrslt2* appears to interfere with the dimorphic transition (Fig. 1). In addition, the disruption of *Mrhog1* or *Mrslt2* leads to restricted vegetative growth and abnormal morphological clones on SMAY plates. This result was unlike the observations in *ΔBbhog1*^26^ and *ΔBbslt2*^27^,^28^. Moreover, similar phenotypic changes have been observed in other fungi^26^,^27^,^28^,^40^, in which the single-gene disruptions increased sensitivity to cell wall damage, high osmolarity, and oxidative stress, as well as showing convoluted colonies. As in other fungi, these results indicated that the CWI and HOG pathways in *M. rileyi* could functionally overlap in responding to cell wall damage, osmoregulation, and oxidative stress^17^,^19^,^24^.

The MS, used as an alternative propagule for biocontrol production, were formed under changing culture conditions^13^. The gens of CWI and HOG pathways were found up-regulated in the comparative transcriptome analysis^10^. As *Mrhog1* and *Mrslt2* are the central of the HOG and CWI pathways, our first RT-qPCR analysis demonstrated that both genes play important roles in regulating MS formation. Iron cations are the critical nutritional factor that induced *M. rileyi* MS differentiation^2^ and, as in *Saccharomyces cerevisiae*^43^, exogenous iron cations could produce oxidative stress (data not shown). Similar to the results in other studies, the two genes are activated to respond to low pH levels and oxidative stress^33^,^44^. The CWI pathway is involved in the maintenance of cell wall integrity^18^ and is the main tolerance mechanism to acidic pH^33^, whereas chitin is a main component of the cell wall^45^. The vegetative growth and MS production defects observed in *ΔMrslt2* mutants suggested a role in morphogenesis. Our observations of enhanced chitin synthesis and compromised melanisation, resulting in breached cell wall integrity, are underlying causes of the morphological defects. Additionally, as in *Verticillium dahliae*^40^, the down-regulation of melanin biosynthetic genes led to lower melanin production levels in *ΔMrhog1* mutants. To survive under changing culture conditions, as described previously, single-gene disruptions interact to regulate cellular multi-stress responses. Furthermore, two transcriptional factors *MrMsn*_*2,4*_, downstream of *Mrhog1,* and *Mrswi4,6,* downstream of *Mrslt2,* were found up-regulated in the comparative transcriptome analysis^10^. Therefore, we chose the *MrMsn*_*2,4*_*, Mrswi4,6,* and the upstream component of CWI and HOG pathways and analysed the transcription level in the *Mrhog1* and *Mrslt2* mutants. This hypothesis was confirmed by the RT-qPCR analysis (Fig. 4). Further experiments are needed to elucidate the cross-talk between the two MAPKs in regulating MS development.

Hog1p and Slt2p are known to contribute to the regulation of pathogenesis in entomopathogenic and phytopathogenic fungi^26^,^27^,^28^,^40^,^46^. In *M. rileyi*, our data indicated that *ΔMrhog1* and *ΔMrslt2* were significantly less pathogenic than WT in both types of insect bioassays. One explanation involves morphogenic defects in the mutant. The inactivation of *Bbhog1* in *B. bassiana*^26^ and *Moslt* in *M. oryzae*^46^ result in defects in appressorium formation. In contrast, *MgHog1* mutants of *Mycosphaerella graminicola*^47^ impair the initiation of infectious germ tubes. Furthermore, in some plant and human fungal pathogens harbouring these mutations are not as virulent due to the defects in invasive growth and conidiation^48^. Our results showed that the mutants were defective in vegetative growth in the hemocoel (data not shown). Another explanation is that fungal pathogens counter the oxidative stress as part of their armoury in the host^49^, and that Hog1p and Slt2p are coordinated to resist oxidative stress^17^,^19^. As in *B. bassiana*, the mutants would be hypersensitive to the oxidative stress^26^,^27^,^28^.

In conclusion, two MAPKs genes were characterized in the entomopathogenic fungus *M. rileyi.* The two genes contributed to stress responses and to regulating MS development by acting in a cooperative manner. This study provides new insight into *M. rileyi* MS development. Further studies are underway to identify the down-stream effectors and mechanism responsible for the *Mrhog1* and *Mrslt2* interaction.

## Materials and Methods

### Microbial strains and growth conditions

The fungal strains were cultured according to previously described methods^12^. *Escherichia coli* DH5α (Invitrogen, Carlsbad, CA, USA) was used for plasmid propagation and cultured in Luria–Bertani medium containing various concentrations of antibiotics based on plasmid resistance. *Agrobacterium tumefaciens* AGL-1 was used in fungal transformations and grown as described by Shao *et al*.^38^.

### Gene cloning and phylogenetic analysis

Based on the partial sequences in the transcriptome library^10^, we designed gene-specific primers for the amplification of cDNA and genomic DNA sequences. Total RNA was extracted using TRIzol^®^ reagent (Invitrogen) and first-stand cDNA was synthesized using SuperScript II Reverse Transcriptase (Invitrogen), following the manufacturer’s instructions. The amino acid sequences were aligned with DNAMAN software (http://www.lynnon.com) and phylogenetic trees were generated using MEGA 6.0 software (http://www.megasoftware.net).

### Generation of *Mrhog1* and *Mrslt2* gene knockouts

The pPZP-Hph-Mrhog1 and pPZP-Hph-Mrslt2 plasmids were proliferated in *E. coli* DH5α and transformed into the WT strain using *Agrobacterium*-mediated transformation^38^. Putative gene disruption mutants were screened on SMAY supplemented with 450 μg/ml Hyg B. To determine the desired recombination event had occurred in the transformants, their genomic DNA was extracted, PCR screening was performed with primers homologous to the *hph* and genomic sequence outside the flank regions (HF/hph-R/HR/hph-F and SF/hph-R/SR/hph-F, respectively) (Table S1) and the amplicons were sequenced. Putative mutants were confirmed by additional Southern blotting. The target fragments were probed with 732-bp and 440-bp probe amplified with primers HosF/HosR and SlsF/SlsR. respectively (Table S1).

### Conidial yield, germination, and vegetative growth

To characterize the role of *Mrhog1* and *Mrslt2* in the yeast-to-hyphae transition, vegetative growth, and conidial development, conidia of indicated strains were harvested and suspended according to previous methods^13^. The conidial suspensions were inoculated onto SMAY plates at 25 °C to record conidial germination rate. Beginning 14 h after inoculation, germination was assessed every 2 h. Three random fields were observed by microscope. In each field, the number of germinated conidia out of 100 conidia was recorded. The average numbers from these visual fields were recorded as the germination frequency. Then, 3 μl of conidial suspensions were dripped onto SMAY plates and cultured under continuous light at 25 °C for 12 days. The colony morphology was examined and images were collected using a digital camera (60-mm Macro lens, Canon Inc., Japan) and microscope.

To analyse abiotic stress tolerance, 3 μl of conidial suspensions were dripped onto SMAY plates supplemented: (I) with NaCl (0.5 mol/l) and KCl (0.5 mol/l) for the salt stress assay; (II) with sorbitol (0.5 mol/l) and glycerinum (1 mol/l) for the osmosensitivity assay; (III) with Congo red (100 μg/ml), Calcofluor (20 μg/ml), and SDS (0.06%) for the cell stress assay; and (IV) with menadione (0.3 mmol/l) and H_2_O_2_ (3 mmol/l) for the oxidative stress assay. All the plates were incubated for 12 days. The conidial yields of each strain were assessed according to previous methods^12^.

### Fungal growth in liquid culture

The mycelia and MS morphologies were observed using digital camera and microscope. After a 6-day incubation, the biomass was quantified in AM and MM cultures. Furthermore, the MS yield was examined in the AM culture. The biomass and MS yields were determined according to previous methods^12^. Meanwhile, a wet-mount of 72-h and 144-h cultures in 0.1% Calcofluor (Fluorescent Brightener 28, Sigma) were viewed and photographed using a fluorescent attachment (Nikon Ni, Nikon Inc., Japan).

### Transcriptional analysis

Three total RNA samples were collected. One collection occurred following the stages of MS development, as described previously^13^. To assess the changing culture conditions effect on *Mrhog1* and *Mrslt2* expression levels, the incubated AM or MM medium was supplemented with exogenous acetic acid, HCl, H_2_O_2_ (3 mmol/l), or iron cation, independently^13^. Then, the mycelia were harvested for another total RNA extraction. To investigate the cooperation of *Mrhog1* and *Mrslt2* to regulate MS differentiation, the WT and mutant strains separately were incubated in liquid AM cultures. After 3 days of incubation, the mycelia were collected and total RNA was extracted.

All of the samples were collected by vacuum filtration and washed twice with sterile water. After digesting with DNase I (TaKaRa), the first-strand cDNA fragment was synthesized. RT-qPCR was performed using a Mini Opticon Real-time PCR System (Bio-Rad) with SYBR Green detection. As internal standard, β-tubulin (*Mrtub*) and translation elongation factor (*Mrtef*) genes were used, and the specific primer pairs are listed in Table S1. The relative quantification of the target gene’s expression level was evaluated using the 2^−ΔΔCt^ method^50^.

### Virulence assays

Virulence was assayed against third-instar *Spodoptera litura* larvae by topically immersion into the conidial suspension (5 μl of a 1 × 10^6^ conidia/ml solution in cottonseed oil) or by injection into a conidial suspension (5 μl of a 1 × 10^6^ conidia/ml solution in sterile water with 0.01% Tween 80). Three replicates comprised of 30 larvae each were tested. For the control, 5 μl of pure cotton seed oil alone or sterile water with 0.01% Tween 80 was applied to or injected into the larvae, respectively. The treated larvae were reared as described previously^12^. The larval mortality rate was recorded every day, and the LT_50_ values were estimated by probit analysis.

### Statistical analysis

All of the experiments were repeated three times. The data obtained were analysed using SPSS 17.0 software. The mean LT_50_ was estimated using SAS version 9.1 software^51^. The graphs were constructed with GraphPad Prism 5 software. The results were recorded as mean ± standard error (SE).

## Additional Information

**How to cite this article**: Song, Z. *et al*. The high osmotic response and cell wall integrity pathways cooperate to regulate morphology, microsclerotia development, and virulence in *Metarhizium rileyi*. *Sci. Rep.* **6**, 38765; doi: 10.1038/srep38765 (2016).

**Publisher's note:** Springer Nature remains neutral with regard to jurisdictional claims in published maps and institutional affiliations.

## Supplementary Material

Supplementary Information

## Figures and Tables

**Table 1:** Analysis of MS yields and biomass values of different strains grown in AM and MM.

Strains	AM culture		MM culture
	MS yields (×10^4^/ml)	Biomass (g/l)	Biomass (g/l)
WT	9.66 ± 0.31 a	48.51 ± 1.24 a	40.67 ± 1.23 a
*ΔH13*	0.005 ± 0.0003 b	14.5 ± 0.47 b	23.62 ± 0.28 b
*ΔH74*	0.006 ± 0.0006 b	13.5 ± 0.51 b	22.15 ± 0.51 b
*ΔS6*	0.38 ± 0.02 c	27.62 ± 0.83 c	31.62 ± 1.48 c
*ΔS11*	0.31 ± 0.03 c	27.85 ± 1.05 c	30.59 ± 1.41 c

Means followed by different lowercase letters within a column are significantly different (Duncan’s multiple range tests).
