# Supplementary material for: The high osmotic response and cell wall integrity pathways cooperate to regulate morphology, microsclerotia development, and virulence in Metarhizium rileyi
Source: Sci Rep. 2016 Dec 12;6:38765. doi: 10.1038/srep38765 (PMC5150533; doi:10.1038/srep38765)
Supplement: Supplementary Information [file srep38765-s1.pdf]

The high osmotic response and cell wall integrity pathways cooperate to regulate morphology, microsclerotia development, and virulence in *Metarhizium rileyi*

Zhangyong Song [a], QiangZhong [a], YoupingYin[a], Ling Shen[a], Yan Li[a], Zhongkang Wang [a,*]

a Chongqing Engineering Research Center for Fungal Insecticide, School of Life Science, Chongqing University, Chongqing 400030, People's Republic of China.

Author 1:

Given name: Zhangyong Family name: Song

Email: szy83529@163.com

Author 2:

Given name: Qiang Family name: Zhong

Email:qiang_zhong@outlook.com

Author 3:

Given name: Youping Family name: Yin

Email:ypy128@vip.sina.com

Author 4:

Given name: Ling Family name: Shen

Email:shen89101@163.com

Author 5:

Given name: Yan Family name: Li

Email: sungq520@yeah.net

Author 6:

Given name: Zhongkang Family name: Wang

Email: w-zk@163.com

Tel/Fax: +86-023-65120489

Table S1 The primers designed for genes analysis

Genes	Name of primer	Sequences (5’-3’)
primers used for cDNA and genomic DNA sequences	*Mrhog1-F*	CATGGGAGCCTTCGGTCTGGTTT
	Mrhog1-R	TGCGCGAGATCAACTCACAAACC
	Mrhog1-gF	CATGGGAGCCTTCGGTCTGGTTT
	Mrhog1-gR	GTGCCTGTCGTATGCCGTCGTTG
	Mrslt2-F	ATGGCCGACCTTCACCTTCA
	Mrslt2-R	CTCCTGGAAGCATCTAGGCCGG
	Mrslt2-gF	TTATCTCCTGGAAGCATCTA
	Mrslt2-gR	ATGGCCGACCTTCACCTTCA
universal primers used for FPNI-PCR	FP1	GTAATACGACTCACTATAGGGCACGCGTGGTNTCGASTWTSGWGTT
	FP2	GTAATACGACTCACTATAGGGCACGCGTGGTNGTCGASWGANAWGAA
	FP3	GTAATACGACTCACTATAGGGCACGCGTGGTWGTGNAGWANCANAGA
	FP4	GTAATACGACTCACTATAGGGCACGCGTGGTAGWGNAGWANCAWAGG
	FP5	GTAATACGACTCACTATAGGGCACGCGTGGTNGTAWAASGTNTSCAA
	FP6	GTAATACGACTCACTATAGGGCACGCGTGGTNGACGASWGANAWGAC
	FP7	GTAATACGACTCACTATAGGGCACGCGTGGTNGACGASWGANAWGAA
	FP8	GTAATACGACTCACTATAGGGCACGCGTGGTGTNCGASWCANAWGTT
	FP9	GTAATACGACTCACTATAGGGCACGCGTGGTNCAGCTWSCTNTSCTT
	FSP1	GTAATACGACTCACTATAGGGC
	FSP2	ACTATAGGGCACGCGTGGT
special primers used for FPNI-PCR	*Mrhog1-F1*	TGTGCTTCCGACAGACAGCCTCCTT
	Mrhog1-F2	GTCGGTGCAGATGGAGATGGACGAG
	Mrhog1-F3	TGACTCGTCGTATCCGCAGAGCAAG
	Mrhog1-R1	TCGGTTCACGACAATGCCTACAGCG
	Mrhog1-R2	GGCAATGTATGTAGGAGTGCGGGGA

	Mrhog1-R3	TCTTGGCGTTTTGGGAGACTTGGTG
	Mrslt2-F1	ACCTTGAAAACCTTTCGTCCCTGAAG
	Mrslt2-F2	GAGAGCCGAACGCAGTAGCTTGAATC
	Mrslt2-F3	AGAAGAATGGGTCGGGTATTTTATGG
	Mrslt2-R1	ATTGTCACTTGCGGGAAGGACGGTA
	Mrslt2-R2	TCGGTAATACCCAGCACGGATAAGAC
	Mrslt2-R3	AGAAGCCCTTTATGCCCCAATGTCG
primers used for flanking sequences	HLF1	cgGAATTCACTTGGGATTTTCTGAACGCACATGG<br>EcoRI site is underlined
	HLR1	ccgCTCGAGAAATGGCGGGATGGCTGGGTTTGAGA<br>XhoI site is underlined
	HRF1	gcTCTAGAAGACAACGACGGCATACGACAGGCAC<br>XbaI site is underlined
	HRR1	cccAAGCTTTGTAGGGCGAACAGGCTCGGGCAAAA<br>HindIII site is underlined
	SLF1	cgGAATTCGAGGGCGCTTAAGGTTGACGGGTTCC<br>EcoRI site is underlined
	SLR1	ccgCTCGAGCGAGAAGAATGGGTCGGGTATTTTATGG<br>XhoI site is underlined
	SRF1	gcTCTAGAGAAGGTAGGGAGGGACGGATGGAAAG<br>XbaI site is underlined
	SRR1	cccAAGCTTAAACACACCCCTTTTCTCTCGCCCC<br>HindIII site is underlined
primers used for PCR screening transformants	HF	GCTGTTTACTTCCGCCATCCATCCCT
	HR	GAGTTTCGAGACGTGACGATCCGCTA
	Ho-OF	CCAGATATTCCGACCTTCAGCCTGTG
	Ho-OR	CCAGCCTCTACATTATGGTAGTCAA
	SF	TGGCGAGAAGTGAAAACGCTGGTAT

	SR	GGGCGCAGGTGCAGGTTCTTGAA
	Sl-OF	CTCGGACAGGGTGCCTATGGTATTG
	Sl-OR	CGCATCTCCCCGACATCGTCAATGA
	hph-F	GCTCTCGCTAAACTCCCCAATGTCA
	hph-R	CATTGACTGGAGCGAGGCGATGTTC
primers used for Southern blotting	HosF	GTTGTCGTTTCTGTTTCATCCCATCC
	HosR	TTTGACTCGTCGTATCCGCAGAGC
	SlsF	GCTCTGAGGTCCTGTAGAAAAGTG
	SlsR	AGAATGGGTCGGGTATTTTATGGC
primers used for RT-qPCR analysis	*Mrhog1-qF*	GCATCGTGACTTGAAGCCTA
	Mrhog1 -qR	GGGCTCGGTAATATCGTGTT
	Mrslt2-qF	TGTGCGGCCTCAAGTATATC
	Mrslt2-qR	CGAGGCCAAAGTCACAGAT
	Mrpbs2-qF	GAGGTCCACATACTGGACGA
	Mrpbs2-qR	CTTGGCATGCTTGACTTTGT
	Mrmsn2,4-qF	AGAGGACCCTTCCAAGACCT
	Mrmsn2,4-qR	GCTTCAGGTGTTCTTGACGA
	Mrmkk1,2-qF	GCCTCCGGTGTATAGCTTGT
	Mrmkk1,2-qR	GTGATCCTACACGAGTGCGT
	Mrswi4,6-qF	CCACCCAGATTCTCAAGGTT
	Mrswi4,6-qR	TGCTCGCCTGTTTGTATCTC
	Mrtef-qF	GTCATCGTCCTCAACCATC
	Mrtef-qR	CAGTCTCAACAGCCTTACC
	Mrtub-qF	GGCAAGGTCGCTATGAAG
	Mrtub-qR	CTGGATGGAGGTAGAGTTAC
	MrpksP-qF	AGGCCTATAAAGGCATGCAA

MrpksP-qR	GCAACTGTCCACCCACATAG
Mrchs1- qF	CATCCGTCAACACCAAAGAC
Mrchs1- qR	GCTGAATAAGGCGACCTCTC
Mrchs2- qF	AATCGGCGACAATTTCTACC
Mrchs2- qR	ATTCGTATCCTGCCTTCCAC
Mrchs4- qF	GTCAATGAGCTCGAAAGTCG
Mrchs4- qR	TCGACGTCATCTACAAAGGC

Fig. S1 Phylogenetic tree interred from Mrhoglp (A) and Mrslt2p (B) protein sequences alignments

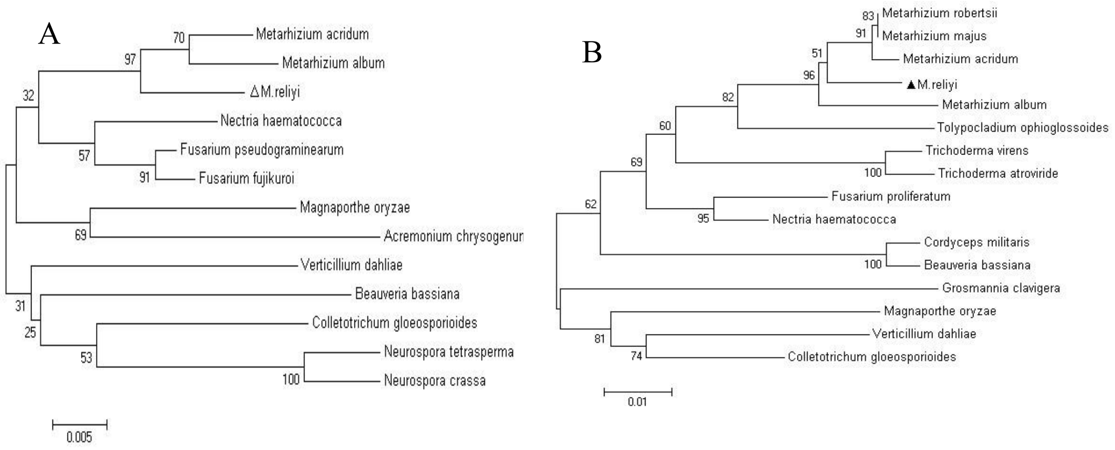

The numbers on the nodes represent the results of bootstrap analyses (1000 replicates) carried out using the neighbor-joining method. The aligned sequences of Mrhoglp protein are from *Acremonium chrysogenum* ATCC 11550 (XP KFH47112.1); *Neurospora tetrasperma* FGSC 2508 (XP 009849980.1); *Neurospora crassa* OR74A (XP 962163.2); *Verticillium dahliae* VdLs.17 (XP 009655080.1); *Colletotrichum gloeosporioides* Nara gc5 (XP 007280298.1); *Fusarium fujikuroi* IMI 58289 (XP CCT64307.1); *Beauveria bassiana* ARSEF 2860 (XP 008598528.1); *Fusarium psedograminearum* CS3096 (XP 009257285.1); *Nectria haematococca* mpVI 77-13-4 (XP 003046679.1); *Magnaporthe oryzae* 70-15 (XP 003714838.1); *Metarhizium acridum* CQMa 102 (XP 007814424.1); *Metarhizium album* ARSEF (XP KHO01547.1). The aligned sequences of Mrslt2p protein are from *Metarhizium robertsii* ARSEF 23 (XP 007819370.2); *Metarhizium majus* ARSEF 297 (XP 014574977.1); *Metarhizium acridum* CQMa102 (XP 007814364.1); *Metarhizium album* ARSEF 1941 (KHN93898.1); *Tolypocladium ophioglossoides* CBS 100239 (KDB88410.1); *Trichoderma virens* Gv29-8 (XP 013959852.1); *Trichoderma atroviride* IMI 206040 (XP 013937734.1); *Fusarium proliferatum* (ABD67163.1); *Nectria haematococca* mpVI 77-13-4 (XP 003050911.1); *Cordyceps militaris* CM01 (XP 006674833.1); *Grosmannia clavigera* kw1407 (XP 006674015.1); *Beauveria bassiana* ARSEF 2860 (XP 008596653.1); *Magnaporthe oryzae* 70-15 (XP 003712437.1); *Verticillium dahliae* VdLs.17 (XP 009651532.1); *Colletotrichum gloeosporioides* Nara gc5 (XP 007285039.1).

Fig. S2 The disruption of *Mrhog1* and *Mrslt2* gene

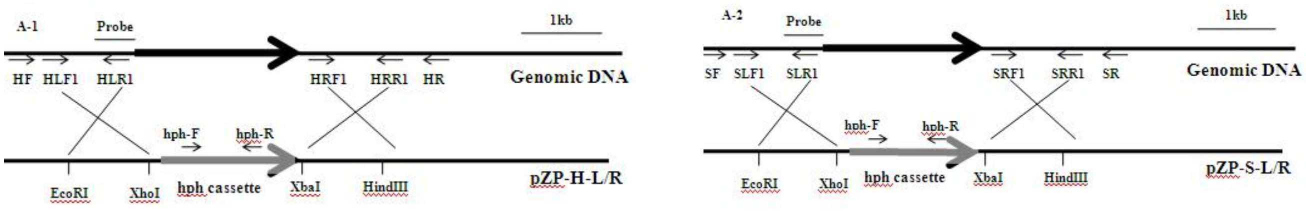

B-1

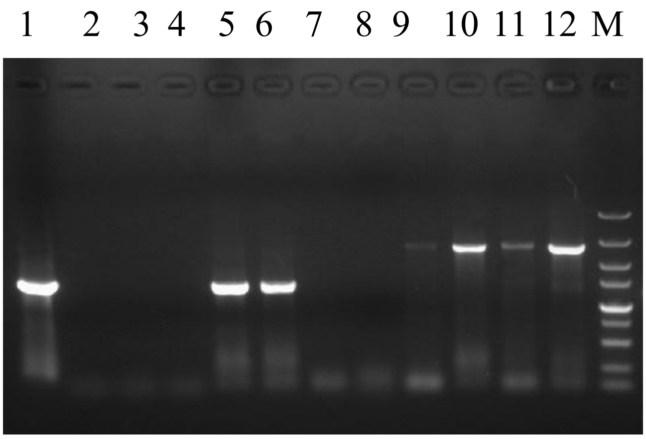

B-2

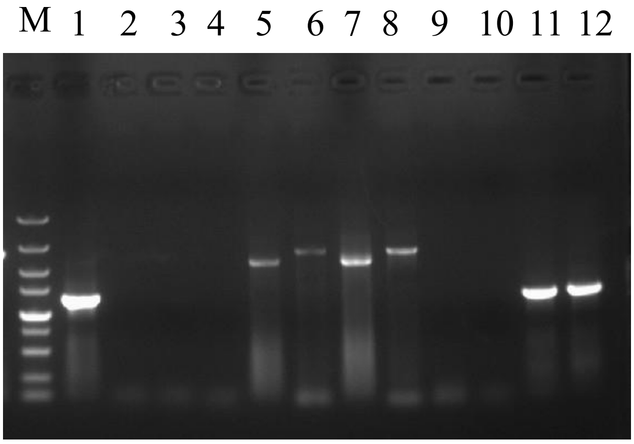

C-1

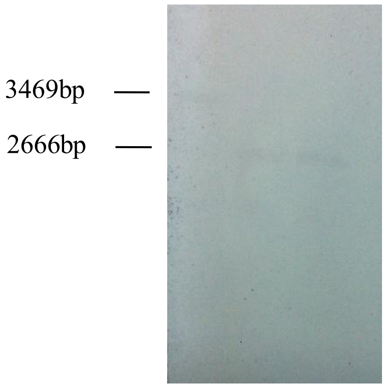

C-2

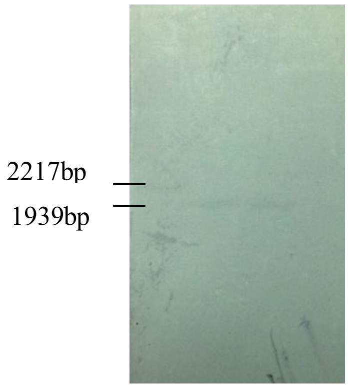

(A) The construct of *Mrhog1* (A-1) and *Mrslt2* (A-2) loci, disruption constructs with *hph* cassette and flanking sequences. Arrows indicate the positions of primers used for PCR screening of deleted mutants and generation of hybridization probes. (B) PCR characterization of the four disrupted mutants. (B-1) PCR characterization of *△MrHog1* strains. PCR screen the ORF (Lane 1-3: 1-wild-type, 2-*△H13*, 3-*△H74*); PCR screen the *hph* (Lane 4-6: 4- wild-type, 5-*△H13*, 6-*△H74*); PCR screen the

hph and genomic sequence outside the flank regions (Lane 7-12: 7-LF-WT, 8-RF-WT, 9-LF-*△H13*, 10-RF-*△H13*, 11-LF-*△H74*, 12-RF-△H74); (B-2) PCR characterization of *△MrSlt2* strains. PCR screen the ORF (Lane 1-3: 1-WT, 2-*△S6*, 3-*△S11*); PCR screen the *hph* and genomic sequence outside the flank regions (Lane 4-9: 4-LF-WT, 9-RF-WT, 5-LF-*△S6*, 6-RF-*△S6*, 7-LF-*△S11*, 8-RF-*△S11*); PCR screen the *hph* (Lane 10-12: 10- wild-type, 11-*△S6*, 12-*△S11*). M, DNA molecular size markers (DL 5000). (C) Southern blotting analysis of *△MrHog1* (C-1) and *△MrSlt2* (C-2) mutants. Genomic DNA was digested with BamHI/SaII and HindIII/XbaI in *△Mrhog1*, *△Mrslt2* and WT isolates. The PCR product obtained with primers HosF/HosR and SlsF/SlsR was used as the probe.

Fig. S3 Germination of conidia from WT, *△MrHog1,* and *△MrSlt2* isolates

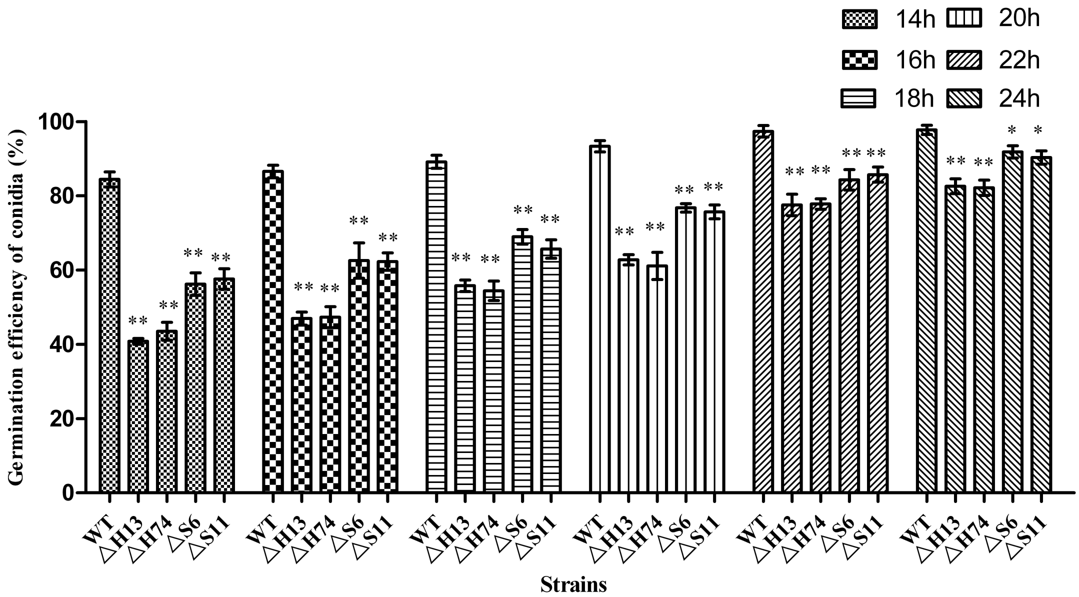

The percentage of conidia germination was determined under a light microscope. Conidium was scored as germinated if the germ-tube length was half in size to that of the conidium. Values were the mean of three independent experiments. Standard error bars indicate variation in measurements.* $P< 0.05$, ** $P< 0.01$, when compared with the results observed at WT.

Fig. S4 The abnormal hyphal morphology of △*Mrslt2* mutants

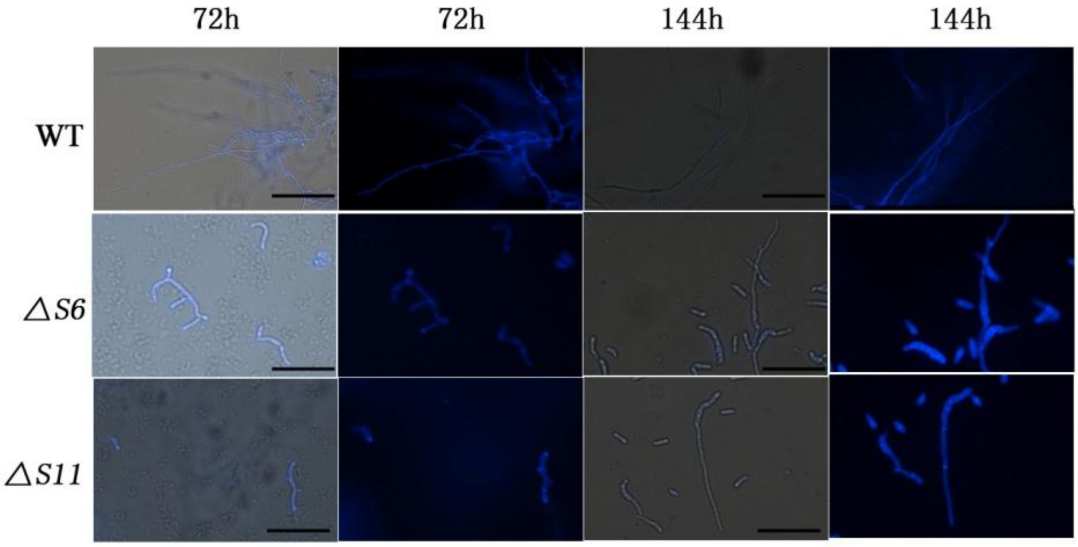

All strains were stained with Calcofluor for chitin distribution and fluorescence was mainly distributed on the apex of hyphae and septa. Mycelial morphology was determined under a microscope after the △*Mrslt2* mutants and WT strains were grown in AM cultures for 3 days. Scale bar: 50 μm.
